# Pro-Atherogenic Inflammatory Mediators in Inflammatory Bowel Disease Patients Increase the Risk of Thrombosis, Coronary Artery Disease, and Myocardial Infarction: A Scientific Dilemma

**DOI:** 10.7759/cureus.10544

**Published:** 2020-09-19

**Authors:** Kaushik Kondubhatla, Ayush Kaushal, Ali Daoud, Hassan Shabbir, Jihan A Mostafa

**Affiliations:** 1 Internal Medicine, California Institute of Behavioral Neurosciences and Psychology, Fairfield, USA; 2 Psychiatry and Behavioral Sciences, California Institute of Behavioral Neurosciences and Psychology, Fairfield, USA; 3 Hematology, California Institute of Behavioral Neurosciences and Psychology, Fairfield, USA

**Keywords:** inflammatory bowel disease, myocardial infarction, inflammation, atherosclerosis, gut microbiota, coronary artery disease

## Abstract

Inflammatory bowel disease (IBD), comprising ulcerative colitis and Crohn’s disease, is characterized by widespread inflammation of the gastrointestinal tract with systemic manifestations. Inflammation is one of the driving forces for the pathogenesis of atherosclerosis and its dreaded complications like myocardial infarction (MI). Yet, the association between IBD and myocardial infarction has not been thoroughly established.

Myocardial infarction in IBD patients was predominantly seen in young women during the active disease process. At the same time, elevated levels of C-reactive protein and other pro-inflammatory markers were observed in both IBD and atherosclerosis. Increasing evidence suggests inflammation inhibits fibrinolysis, expresses procoagulants, and suppresses anticoagulants promoting thrombosis formation. Moreover, the alteration of gut microbiota impacts the pathogenesis of inflammation and predisposes one to ischemic heart disease.

Accordingly, all IBD patients should be screened and counseled on lifestyle modifications for the traditional risk factors of atherosclerosis. Future researchers should consider conducting more clinical trials on anti-inflammatory medication targeting atherosclerosis and therapeutics, while targeting the gut microbiota to reverse the inflammatory atherosclerotic process.

## Introduction and background

Inflammatory bowel disease (IBD) is characterized by widespread chronic inflammation of the gastrointestinal tract. The two central pathologies that compose IBD include ulcerative colitis and Crohn’s disease. Patients are typically diagnosed between the ages of 15-35 years, routinely present with abdominal pain, diarrhea, bloating, rectal bleeding, abdominal cramps, and weight loss [1,2]. The disease process contains a wide range of outcomes that include complete remission with minimal treatment to long-lasting flares and relapses, despite active management. Studies report that within the first eight years of diagnosis, 20% of patients with Crohn’s disease relapse yearly, and 67% of the patients oscillate between remission and relapse. The same literature suggests there is a 9-21% cumulative risk in ulcerative colitis patients that they will relapse to a level where a colectomy would be life-saving [3].

Inflammatory bowel disease is considered idiopathic in nature, but factors that predispose the condition include immune response, genetic, environmental, and microbial factors [4]. The chronic inflammation in IBD is distinguished by widespread elevation in C-reactive protein, homocysteine, fecal calprotectin, and sedimentation rate [3,5]. 

At the same time, studies have shown that inflammation plays a critical role in the development of atherosclerosis. This process is regulated by an interaction between pro-atherogenic inflammatory and anti-inflammatory mediators, which can lead to the development of thrombosis, and increase susceptibility to coronary artery disease (CAD). Proatherogenic inflammatory mediators consist of T-helper cell type 1 and lymphocyte B2 cells, while regulatory T-cells and types of T-helper 2 related cytokines illustrated an anti-atherogenic effect. Long-standing elevation of levels of C-reactive protein, interleukin 6 (IL-6), activated protein C is now widely considered independent risk factors for coronary artery disease [6,7].

Many experts widely document that chronic inflammation increased the incidence of cardiovascular manifestations. But, the association between IBD and myocardial infarction (MI) has not been entirely established. [8,9].

Recent studies have illustrated mixed results: Some showed an association [10], while others showed no significant association [11]. Some studies have portrayed an increased association between IBD with MI due to inflammation as an essential risk factor rather than the conventional ones like smoking, hyperlipidemia, hypertension, and diabetes [6]. 

It is essential to establish the association between the two conditions so that measures can be taken to start preventing the disease. The purpose of this review is to examine the current literature regarding inflammatory bowel disease and myocardial infarction and establish the relationship between the two conditions.

## Review

Although it predominantly presents in Western societies, cases of inflammatory bowel disease have risen worldwide. Ulcerative colitis and Crohn’s disease are grouped in the same disease process; however, subtle differences exist between the two inflammatory disorders, as can be seen in Table 1.

**Table 1:** Ulcerative Colitis vs Crohn’s Disease

Features	Ulcerative Colitis	Crohn’s Disease
Location	Common in colon and rectum	Common in distal Ileum, can occur anywhere in the gastrointestinal tract
Pathogenesis	Inflammation occurs uniform/continuous in the affected areas	Inflammation occurs irregularly in patches known as Skip lesions
Clinical Features	Bloody diarrhea, weight loss, lower left abdominal pain	Crampy pain in the right abdomen, diarrhea, weight loss
Macroscopic features	Ulcerated pseudopolyps, thin colon wall	Aphthous ulcers, cobblestone appearance, creeping fat, Thick wall
Microscopic features	Crypt abscesses	Non-caseating granuloma
Complications	Toxic megacolon, colon cancer	Strictures, abscesses, sinus tract and fistula formation and Obstruction
Extraintestinal manifestations	Arthritis, primary sclerosing cholangitis, pyoderma gangerosum, erythema nodosom	Anemia, nephrolithiasis, scleritis, episcleritis, cholelithiasis, thromboembolism, erythema nodosom.

According to a study assessing the global and national burden of Inflammatory bowel disease, the prevalence of IBD in the United States of America was reported to range from 252 to 439 cases per 100,000 population. The evidence of IBD revealed to be more prevalent in females compared to males. Due to the chronic nature and low mortality of the disease it’s prevalence is high and more complications are encountered. [12].

Genetic, environmental factors and microbial factors in IBD and cardiovascular disease

Although inflammatory bowel disease (IBD) is widely accepted as a chronic inflammatory process, the exact cause is unknown. It is stated that there are interactions between a wide array of environmental, host, microbial, and genetic factors [13].

Role of Genetic Factors

Analyzing IBD patients’ genetic material, illustrated that there is a distinctive locus that regulates intracellular bacteria-killing, innate immunity, and adaptive immune responses. A lesion in that locus could impair the immune system, upregulate cytokines, and disrupt bacteria clearance contributing to the chronic inflammatory process [14].

Role of Environmental Factors

Environmental factors play a significant role in the disease process. Studies confirm smoking has a protective effect in patients with ulcerative colitis, as these patients reporting lower rates of relapse, and hospitalizations; however, smoking showed a completely inverse association in patients with Chron’s disease. Cigarette smoking predisposed patients to a more complicated disease course despite aggressive therapy and an increased requirement for surgery. Hence, smoking cessation is considered one of the most critical lifestyle modifications recommended to Crohn’s disease patients. Although it is crucial to establish smoking has no therapeutic effect in patients with ulcerative colitis, patients with a prior habit should not be discouraged from quitting due to the benefits outweighing the risk. Other environmental factors studied include vitamin D deficiency and increased stress, which showed an increased susceptibility to develop inflammatory bowel disease. [3,15-17]. 

Also, body mass index (BMI) correlated with the active disease process in Chrons disease, as an increase in extraintestinal manifestations was observed in patients with high BMI, suggesting that chronic inflammation associated with obesity may play a role in the disease process. At the same time, obesity and smoking are two of the most notorious risk factors for myocardial infarction. Accordingly, they both contribute to the active disease process for Inflammatory bowel disease, insinuating an indirect association between the two-disease process.

Role of the Gut Microbiome

The gut microbiome, which develops and stabilizes in the second week of life, has around 1150 bacterial organisms that have shown multiple pathological changes throughout the IBD process, termed as dysbiosis. The predominant bacteria in healthy adults arise from the phyla: Bacteroidetes and Firmicutes. These, along with Actinobacteria and Proteobacteria, comprise 99% of the intestinal bacteria. The gut microbiota provides many vital functions such as nutrition, metabolism, contributing to bacterial defense, and immune function. The bacteria regulate the development of various types of T-cells, like regulatory T-cells and Th17 cells. (T-helper 17 cells) Regulatory T-cells have shown anti-inflammatory properties by suppressing different types of immune cells in the immune system. T-helper 17 cells (Th17) cells are characterized by their ability to produce various interleukins (IL) such as IL-17A, IL-17F, IL-21, and IL-22 in the gut microbiota. A decrease in bacteria with anti-inflammatory properties has been the culprit in patients with IBD, evidence stemming from comparing the gut microbiota of an IBD patient to a healthy person [18-20].

There was an abundance of Proteobacteria such as E. coli and Bacteriodetes reported, while anti-inflammatory organisms like Firmicutes are decreased. The surplus bacteria with adhering properties like E. coli contribute to alteration in the gut bacteria, instigating an immune response leading to extensive inflammation. Metabolites such as hydrogen-sulfate produced from bacteria damage the intestine’s epithelial cells disrupting the barrier function, which increases intestinal permeability and inducing more mucosal inflammation. When the barrier function is compromised, alterations in the tight epithelial junctions can enhance the activity of inflammatory cytokines such as tumour necrosis factor alpha (TNF-alpha), interferon-gamma, and interleukins IL-13 resulting in systemic endotoxemia [3,18-20].

Studies show several disorders affecting the heart, such as obesity and metabolic syndrome linked to alteration or deficiency in the gut microbiota. An increased amount of Firmicutes species have been linked to an obese person compared to a lean person. Depending on the bacteria present, a number of patients have weight gain and elevated blood sugars compared to someone consuming the same number of calories [21].

Obesity and metabolic syndrome predispose to atherosclerosis and coronary artery disease (CAD) representing another indirect association between inflammatory bowel disease and myocardial infarction. Hypercholesterolemia and hypertension are the two most important etiologies triggering MI; therefore, it’s important to consider screening for the specific markers that predict CAD and start prophylaxis to prolong or completely prevent MI.

Consequences of inflammation in IBD activity on the cardiovascular system 

*Pathophysiology of inflammation in IBD activity* 

Dysfunction in the innate and adaptive immune responses have been well established in inflammatory bowel disease. The intestinal lamina propria houses a complex immune cell network that maintains the homeostasis required to fight pathogens that enter the microbiota. During the disease process, the lamina propria is invaded by innate immune cells, which include neutrophils, macrophages, natural killer cells, and adaptive immune cells like B-cells and T-cells. Innate immunity initiates when an antigen gets recognized by Toll-like receptors and nucleotide-binding and oligomerization domain (NOD)-like receptors located on the cytoplasm. Studies have shown the expression and function of both receptors are defective during the disease process. NOD2 mutations, commonly associated with Crohn’s disease, showed a weak ability to respond to lipopolysaccharide, which is said to contribute to disease severity. Evidence suggests that leads to decreased activation of nuclear factor kappa B (NF-κB), which leads to reduced anti-bacterial defense and increased microbial invasion and cause immune tolerance [3].

Interleukins (IL)-23, a cytokine that influences innate and adaptive immunity, acts as an early defense mechanism against bacteria by inducing Th17 cells; polymorphisms of IL-23 revealed to be associated with the chronic inflammation seen in inflammatory bowel disease. The adaptive immune response consists of various types of T-helper cells (Th1, Th2, and Th17) and regulatory T-cells, which are branches of CD4 T-cells secreting cytokines to maintain the homeostasis of the intestinal lumen. These branches of cells are critical against preventing entry to pathogens; however, reports show that the overexpression of these cells can contribute to the widespread inflammation seen in the disease process [22,23].

All findings come back to the importance of gut microbiota and inflammation for the pathogenesis of inflammatory bowel disease. Evidence suggests that altered gut microbiota can increase the permeability of the intestine, triggering inflammation as the etiology shared by both conditions. As mentioned, obesity and metabolic syndrome due to the diseased gut microbiota both contribute to the development of coronary artery disease.

Atherosclerotic Plaques in Response to the Inflammatory Process

Atherosclerosis is an inflammatory process responsible for many complications, including coronary artery disease, myocardial infarction, and peripheral arterial disease. Immune and adaptive cells regulate the process through proatherogenic inflammatory and anti-atherogenic mediators. Mononuclear phagocytes are present in all stages of the disease, illustrating the evidence to associate inflammation and atherosclerosis [7,24].

Studies thoroughly document no single marker can alone identify or diagnose inflammatory bowel disease. The serological markers best correlated with the inflammatory process include C-reactive protein (CRP), tumor necrosis factor-alpha (TNFα), immunoglobulins (IgG, IgM), vascular endothelial growth factor (VEGF), interleukin 1 (IL-1), and antineutrophil cytoplasmic antibody. The inflammatory cytokines after activation, regulate atherosclerotic plaque formation through increased oxidative stress, mononuclear phagocyte accumulation, and endothelial dysfunction [14].

Several studies indicate C-reactive protein (CRP), an acute-phase reactant, and high sensitivity C-reactive protein (hs-CRP) are strongly associated with an increased risk of myocardial infarction. According to a meta-analysis done by Emerging Risk Factors Collaboration, CRP concentration showed a positive linear association with several conventional risk factors assessing for risk of vascular disease. Additional studies indicate increased CRP levels equated with relapse of the disease process. High CRP, with increased stool frequency and low serum albumin levels, was associated with a deterioration of ulcerative colitis and failure in medical therapy. At the same time, the American Heart Association and American College of Cardiology considered hs-CRP ≥2.0 mg/L as a strong risk factor for coronary heart disease, and recommend lifestyle modifications to reduce that the risk. In the intestinal immune system, macrophages have anti-inflammatory properties that remove bacteria with minimal damage. Disruption of this process leads to inflammation, impaired immune response, and increased growth of bacteria with filamentous and flagellated features. The flagellated bacteria begin an exaggerated inflammatory episode disrupting the intestinal mucosal barrier. As a result, lipopolysaccharide can cross the intestinal barrier triggering atherosclerosis [3,14, 25, 26]. 

Inflammation, Thrombosis and Coronary Disease

Immune-mediated disorders have been linked to increased cardiovascular events. Proposed mechanisms include autoantibodies, autoantigens, and inflammation triggering the synthesis and rupture of atherosclerotic plaque [8,27-28].

Studies show inflammation inhibits fibrinolysis, expresses procoagulants, and suppresses anticoagulants promoting thrombosis formation. Arterial and venous thromboembolism as a result of inflammatory bowel disease or flares has been well documented. Deep vein thrombosis and pulmonary embolism are the most prevalent complications arising from the hypercoagulable state of IBD, causing a high fatality. Endothelial injury, alterations in the clotting system, and venous stasis are the proposed mechanisms for thrombogenesis. Elevated plasma homocysteine levels have been seen in IBD patients. Homocysteine has been found to contribute atheroma and thrombosis formation once it binds to the endothelium [29-31]. 

Inflammation being the driving force for IBD’s hypercoagulable state and atherosclerosis provides a lot of insight and further evidence that IBD can predispose to Myocardial Infarction.

Common risk factors for IBD, MI, and coronary disease

Many meta-analyses and cohort studies have been conducted globally to determine the association between IBD and MI. According to a study using population data from Finland, there was a notable increase in myocardial infarction in patients with IBD [31]. 

A meta-analysis conducted by Feng and his team in 2017 demonstrated an increase in the risk of ischemic heart disease in patients with IBD, especially Crohn’s disease [32]. Data also suggests that patients will experience increased atherosclerosis rates at a young age with or without the traditional risk factors that usually predispose to myocardial Infarction.

Most literature suggests trapped oxidized lipoproteins in the endothelium activate cells leading to the accumulation of chemokines and cell adhesion molecules, which signal monocytes to marginate and transform to foam cells by accumulating lipids which form the plaque. The same process was seen when microvascular endothelial cells were isolated from the intestine, suggesting shared environmental factors may predispose to the disease process of atherosclerosis leading to MI and IBD. A large retrospective study evaluating the efficacy of statins against inflammation illustrated an 18% decrease in the initiation of corticosteroid therapy. Routinely prescribed cardiovascular medications like statins showed decreased disease activity in IBD, suggesting further evidence on the link between IBD and MI [8,10,33-36]. 

Not all literature accepts the association between IBD and MI. According to a study using the nationwide inpatient sample, patients with IBD were less likely to be hospitalized with MI compared to the general population. The frequency of acute MI in IBD was compared to the rate it occurs in other immune-mediated disorders. A lower frequency was noted in IBD, indicating a difference in pathogenesis [37].

Low-density lipoprotein (LDL) is a well-established marker for predicting CAD; in contrast, high-density lipoprotein (HDL) is known to exert anti-atherogenic and anti-inflammatory effects to prevent it. It is said that HDL can become proatherogenic due to inflammation, diabetes, and oxidative stress [38].

According to a study done by Mitra et al. in 2015 indicated asymptomatic atherosclerotic plaques were associated with an increase of microorganisms of the gut microbiome, such as Bacteriodaceae. Streptococcacea, and Micrococcacea, while symptomatic plaques illustrated an abundance of Helicobacteracea and Neisseriaceae [39]. 

Recent studies depicted that alteration of gut microbiota and its metabolites predisposed to hypertension and vascular pathology, as well as its ability to cause hypercholesteremia. Trimethylamine N-oxide (TMAO), a metabolite produced by the microbes, illustrated a positive correlation influencing the pathogenesis of MI. TMAO inhibits reverse cholesterol transport and accumulates foam cells (macrophages), which accelerate atherosclerosis [38,40,41]. According to a study by Li et al. in 2017, TMAO levels in acute coronary syndrome were considered a short term and long term predictor of cardiovascular pathology [42].

This study does have some limitations. Only literature from the year 2000 was used to contribute to this review. A few articles contained only abstracts without the full text, depriving some specific examples of studies. Evidence in recent years is starting to endorse the association gut microbiota and pathogenesis of CAD. Although human trials were done, some of the studies showing the association between IBD and MI is based on experiments done on animals like mice. Most human research consisted of cross-sectional studies rather than longitudinal cohort studies. Mouse models usually analyzed one specific gene or function, but when it comes to humans, disruption of multiple genes and functions contribute to the disease process. Hence future researchers should focus on conducting additional cohort studies and clinical trials targeting the diseased gut microbiota of humans. 

## Conclusions

After reviewing the literature, much evidence suggests inflammatory bowel disease (IBD) can predispose to myocardial infarction. Physicians should emphasize screening all young patients, particularly women under the age of 50, with IBD, as they have shown a higher risk of developing MI in IBD compared to men. Physicians should consider prescribing statins during the active disease process and counsel patients on lifestyle modifications to prevent the traditional risk factors for coronary artery disease. 

Future research should focus on clinical trials and population studies studying the diseased gut microbiota in patients. Evidence points to the altered gut microbiota, increasing intestinal permeability triggering inflammation as the pathogenesis shared by both conditions. Scientists should consider future research on performing more clinical trials in patients with anti-inflammatory medications to assess therapeutic efficacy in atherosclerosis. Drugs targeting diseased gut microbiota should be thoroughly studied as they could be the future in the management of atherosclerosis and inflammatory bowel disease.
